# An Earliest Endosymbiont, *Wolbachia massiliensis* sp. nov., Strain PL13 from the Bed Bug (*Cimex hemipterus*), Type Strain of a New Supergroup T

**DOI:** 10.3390/ijms21218064

**Published:** 2020-10-29

**Authors:** Younes Laidoudi, Anthony Levasseur, Hacène Medkour, Mossaab Maaloum, Mariem Ben Khedher, Masse Sambou, Hubert Bassene, Bernard Davoust, Florence Fenollar, Didier Raoult, Oleg Mediannikov

**Affiliations:** 1 Aix Marseille Univ, IRD, AP-HM, MEPHI, 13385 Marseille, France; younes.laidoudi@yahoo.com (Y.L.); anthony.levasseur@univ-amu.fr (A.L.); hacenevet1990@yahoo.fr (H.M.); mariem.ben-khedher@etu.univ-amu.fr (M.B.K.); bernard.davoust@gmail.com (B.D.); didier.raoult@gmail.com (D.R.); 2 IHU Méditerranée Infection, 13385 Marseille, France; florence.fenollar@univ-amu.fr; 3 Laboratory of Biology and Health, Faculty of Sciences Ben M’sik, Hassan II University, Sidi Othmane, Casablanca 7955, Morocco; momossaab@gmail.com; 4 Aix Marseille Univ, IRD, AP-HM, SSA, VITROME, Marseille, France, 13385 Marseille, France; massezorro1@gmail.com (M.S.); Hubert.Bassene@ird.fr (H.B.); 5 Campus Commun UCAD-IRD of Hann, Dakar 10200, Senegal

**Keywords:** *Wolbachia*, bedbug, *Cimex hemipterus*, isolation, culture, genomics, B-vitamins

## Abstract

The symbiotic *Wolbachia* are the most sophisticated mutualistic bacterium among all insect-associated microbiota. *Wolbachia*-insect relationship fluctuates from the simple facultative/parasitic to an obligate nutritional-mutualistic association as it was the case of the bedbug-*Wolbachia* from *Cimex* *lectularius*. Understanding this association may help in the control of associated arthropods. Genomic data have proven to be reliable tools in resolving some aspects of these symbiotic associations. Although, *Wolbachia* appear to be fastidious or uncultivated bacteria which strongly limited their study. Here we proposed *Drosophila* S2 cell line for the isolation and culture model to study *Wolbachia* strains. We therefore isolated and characterized a novel *Wolbachia* strain associated with the bedbug *Cimex* *hemipterus*, designated as *wChem* strain PL13, and proposed *Wolbachia* *massiliensis* sp. nov. strain *wChem*-PL13 a type strain of this new species from new supergroup T. Phylogenetically, T-supergroup was close to F and S-supergroups from insects and D-supergroup from filarial nematodes. We determined the 1,291,339-bp genome of *wChem*-PL13, which was the smallest insect-associated *Wolbachia* genomes. Overall, the *wChem* genome shared 50% of protein coding genes with the other insect-associated facultative *Wolbachia* strains. These findings highlight the diversity of *Wolbachia* genotypes as well as the *Wolbachia*-host relationship among Cimicinae subfamily. The *wChem* provides folate and riboflavin vitamins on which the host depends, while the bacteria had a limited translation mechanism suggesting its strong dependence to its hosts. However, the clear-cut distinction between mutualism and parasitism of the *wChem* in *C*. *hemipterus* cannot be yet ruled out.

## 1. Introduction

Bacteria of the genus *Wolbachia* represent the most successful symbiotic bacteria in the terrestrial ecosystem. Gram-negative bacteria of the family Anaplasmataceae in the order Rickettsiales, they are obligatory intracellular endosymbionts of several invertebrate taxa, Arthropoda and Nematoda. Till now, only one species, *Wolbachia pipientis*, has been axenically isolated and officially described [1]. *Wolbachia* are genetically diverse, as are the interactions with their hosts [2,3,4,5,6]. Most of *Wolbachia* genotypes, representing microbiologically separate species, were never isolated in pure culture. Currently, there is a general consensus to classify all genotypes in monophyletic lineage groups or supergroups from A to R, with a new supergroup “S” recently identified from the pseudoscorpion *Atemnus politus* [7]. The supergroups C, D and J infect exclusively filarial nematodes (Onchocercidae) [8,9,10]. Supergroup L exclusively contains plant parasitic nematodes (Pratylenchidae) [11,12]. *Wolbachia* supergroup F is the only clade composed by strains that infecting arthropods and some infecting filarial nematodes [13,14]. This includes especially hematophagous arthropods, such as biting Diptera and Hemiptera, fleas, lice and parasitic mites [15,16,17,18,19,20,21,22,23,24,25,26]. Recently, a novel strain of *Wolbachia* belonging to the supergroup F was isolated in *Ixodes scapularis* cells from a pool of *Ctenocephalides* sp. cat fleas [3].

The problems in *Wolbachia* taxonomy are evident. Different *Wolbachia* genotypes correspond clearly to different species both genetically and biologically [27]. Genetic distances among *Wolbachia* supergroups are huge, moreover, the same genotype may infect different insect species. Although attempts to classify *Wolbachia* in different genera were already done [28], but not widely accepted, mostly because of the difficulties in strain isolation [29]. Until recently, only few *Wolbachia* strains from clades A and B were known in axenic culture. The recent isolation of *Wolbachia* from *Ctenocephalides felis* in tick cell line is a rare example of a successful isolation of *Wolbachia* strain in cell culture [3].

Five distinct reproductive manipulations are induced in *Wolbachia* arthropod hosts: cytoplasmic incompatibility (CI), parthenogenesis induction, killing of male, feminization and meiotic drive, all of which promote its spread by reducing competition for resources from males (a dead-end host) or by imposing an adaptation cost on uninfected females [30,31,32,33]. In some cases, *Wolbachia* form obligate and apparently beneficial relationships with their hosts [34,35]. For blood-feeding Diptera, the CI is the common phenotype [3,17,22]. In addition, *Wolbachia* has long been of applied interest in biological control for vector-borne disease control, *Wolbachia* symbiosis can be harnessed for vector control as well as the potential to combine the sterile insect technique and *Wolbachia*-based approaches for the enhancement of population suppression programs [36]. In filarial nematode diseases such as onchocerciasis and lymphatic filariasis, the use of antibiotics for *Wolbachia* elimination can safely clear adult worm infections [37]. Furthermore, several control programs releasing *Wolbachia*-infected *Aedes aegypti* to reduce the transmission of dengue and other arboviruses [38], because of *Wolbachia* infections can suppress the dissemination and transmission of pathogens in insects, especially when transinfected into a novel host [39]. 

Bed bugs are obligatory hematophagous insects with hemimetabolous development from egg to adult through five nymphal stages (instars), each of which requires a blood-meal to molt to the next stage [40]. They have re-emerged over the last decades worldwide where they may cause problems in housing facilities, public facilities, and residential complexes. In economically advanced countries, they are a serious public health. Bed bug infestations have been reported to have physical and psychological effects in humans. In addition, despite isolation of several pathogens, found in the bed bug body, they have not been confirmed as a vector of pathogens to humans [41,42].

Infections with *Wolbachia* species of F supergroup seem to be common in the Cimicinae subfamily (*Cimex* and *Oeciacus* genera) [40,42]. *Wolbachia*’s relationship with *Cimex lectularius* presumably evolved from a facultative association to obligate mutualism where the bacteria garner protection and nutrients within their host in exchange for supplementing the host’s nutritional needs [20,22,43]. It was suggested new hypotheses about the coordination of *Wolbachia* growth and regression with its host’s physiology and endocrine events [40].

Here, on the basis of taxono-genomic approach, we present the description of *Wolbachia massiliensis* strain PL13 (CSURP2929), a new species of the genus *Wolbachia* belonging to a new Supergroup T, isolated from wild bed bugs *Cimex hemipterus* from Senegal. Its growth condition as well as complete annotated genome are detailed.

## 2. Results

### 2.1. Isolation, Culture, and Description of the Bacterium

**Figure 1:**
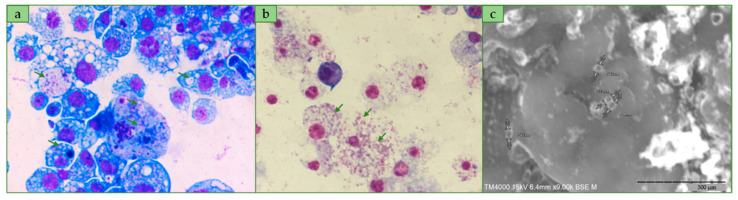
*wChem* PL13 grown (arrowed) in S2 cell-line. (**a**,**b**): Diff-Quik staining, ×1500 showing the intravacuolar location and bacterial load of the *wChem* PL13 strain, respectively. (**c**): scanning microscopic examination of the *wChem* PL13 strain.

Four of ten inoculated shell vials produced morphologically identical bacterial strains. Intracellular growth of bacteria were observed beginning from 14th day post inoculation. The 16S rRNA sequencing revealed the homogeneity of all isolated strains which belonged to the genus *Wolbachia* according to the blast analysis. One strain, designated *Wolbachia* sp. *wChem* PL13, was then selected for the following investigations and characterization. *wChem* PL13 was best visualized by the Diff-Quick staining while on the Gimenez and Gram staining the bacteria stained poorly, but always appeared to be gram negative. The bacteria appeared as small cocci not connected with each other inside intracellular vacuoles but not in the cytoplasm nor in the nucleus (Figure 1a). Therefore, the infected cells showed several vacuoles of different size according to the bacterial load within these latter (Figure 1a). Heavy infected cells were often disrupted during centrifugation using a Cytospin (Thermo Shandon) centrifuge as revealed by subsequent staining suggesting the fragility and death of S2 cells at hight infection levels (Figure 1b). Meanwhile, the infected cells continued to be able to multiply without there being any obvious cytopathic effect. Scanning microscopic examination showed that the bacterium present in the extracellular environment following cell lysis during cytocentrifugation have an average dimension of 570 nm (range: 530 to 615 nm). The bacteria present a regular form of a small cocci (Figure 1c).

The strain *wChem* PL13 was successfully propagated throughout both S2 and C6/36 cell lines. The bacterial growth was better within S2 cell lines at 28 °C according to the qPCR results (Table S1). The difference between S2 and C6/36 cell lines in terms of bacterial load has become significant after three weeks of co-culture, while the difference between culture conditions (temperatures) has become significant after two weeks favorably to cultures maintained at 28 °C for both cell lines (Table S1). In term of speed of growth, the load of the *wChem* PL13 strain has become significantly observable after two and three weeks of co-culture with S2 cells maintained at 28 °C and room temperature respectively, and after three weeks within C6/36 cells from both conditions (28 °C and room temperature) (Table S1).

During the whole processes of the purification, no loss of the bacterium and the sonication step induced only the lysis of cells but not that of the bacteria (Figure S1a), while the gradient density purification provided an integrated bacterium with high density concentration (Figure S1b). Accordingly, the qPCR results indicated the maintenance of bacterial load during all purification steps. At the end of the purification, the bacteria were highly concentrated which gave the Ct value of 11.23 corresponding to 42.86 ng/µL.

### 2.2. Genome Sequencing, Annotation and Genomic Comparison

**Figure 2:**
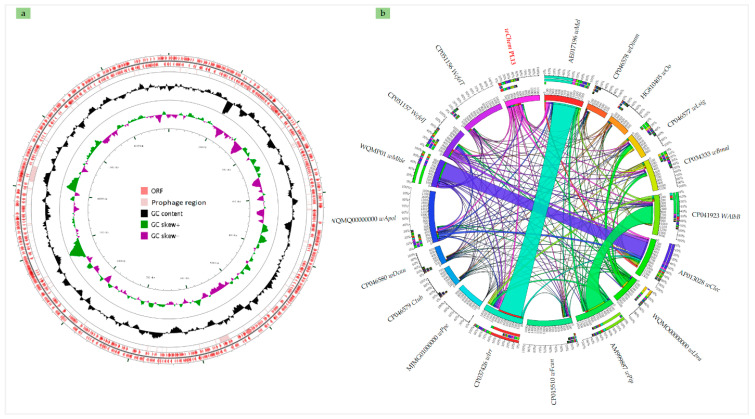
The complete chromosome of the *Wolbachia* sp. *wChem* PL13. (**a**): circular map showing the annotation of the whole genome. (**b**): rhizome showing gene sharing between *Wolbachia* genomes.

De novo assembly based on Illumina and MinION rids (Figure S2) gave a genome sequence from the *wChem* PL13 constructed by one contig of 1,291,339 with a G + C content of 35.4% (Figure 2a). We identified a total of 1226 predicted protein-coding genes, in addition to 3 complete rRNA operons, 32 tRNAs and 1 tmRNA. Comparison of these genomic data with those from of the other *Wolbachia* supergroups showed that the genome of *wChem* PL13 is close to those encountered in other insects (Table 1). The strain *wChem* PL13 showed the presence of two prophage regions of 14.5 Kbp and 23.4 Kbp (Figure 2a and Figure S3). Blast analysis revealed that the *wChem* PL13 prophages shared up to 19% to 58% with an identity ranged from 76.76% to 87.25% with those of other insect-associated *Wolbachia* such as *wDmel*, *Wolbachia* of *D. melanogaster* from supergroup A (AE017196) and *wFcan*, *Wolbachia* of *F. candida* from supergroup E (CP015510). However, up to 50% of the predicted protein-coding genes from the PL13 genome were shared with other *Wolbachia* supergroups (Figure 2b).

**Figure 3:**
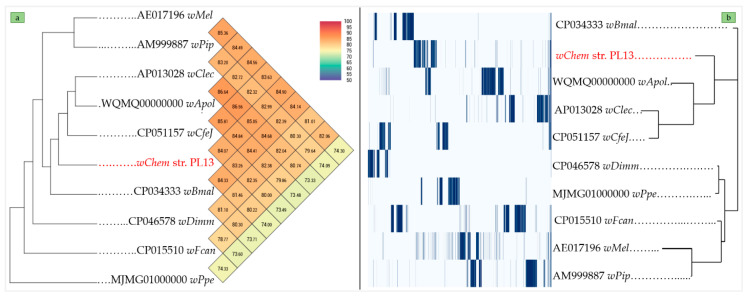
(**a**). heatmap generated with Ortho-ANI values between the *wChem* PL13 strain and the other *Wolbachia* supergroups. (**b**). pan-genome analysis of *wChem* PL13 strain based on the maximum likelihood tree from the accessory genome elements (right). The presence (blue) and absence (white) of accessory genome elements are presented on the left matrix.

Ortho-ANI values (Figure 3a) ranged from 74.63% with wPpe, *Wolbachia* supergroup L from *P. penetrans* (MJMG01000000) to 84.82% with *wClec*, *Wolbachia* supergroup F from *C. lectularius* (AP013028). The pangenome analysis of the *wChem* PL13 strain showed a total of 11,402 clusters genes distributed as follows: (Core genes = 0), (Soft core genes = 0), (Shell genes = 210) and (Cloud genes = 11,192), respectively. The Ortho-ANI and the pangenome trees were clearly congruent (Figure 3a,b), where the *wChem* PL13 strain clustered with wApolK5, *Wolbachia* supergroup S from *A. politus* (WQMQ00000000), *wClec*, *Wolbachia* supergroup F from *C. lectularius* (AP013028) and *wCfeJ*, an undescribed *Wolbachia* supergroup from *C. felis* (CP051157).

Genomic comparison of the *wChem* PL13 strain with the other *Wolbachia* supergroups using Digital DNA-DNA hybridization values (dDDH) are reported in Table 2. For the strain PL13, these values ranged from 19.8% with *Ctub*, *Wolbachia* supergroup J from *C. tuberocauda* (CP046579) to 30% with *wApolK*5, *Wolbachia* supergroup S from *A. politus* (WQMQ00000000).

### 2.3. B-Vitamin Synthesis Patterns in the wChem PL13 and Other Wolbachia Genomes

**Figure 4:**
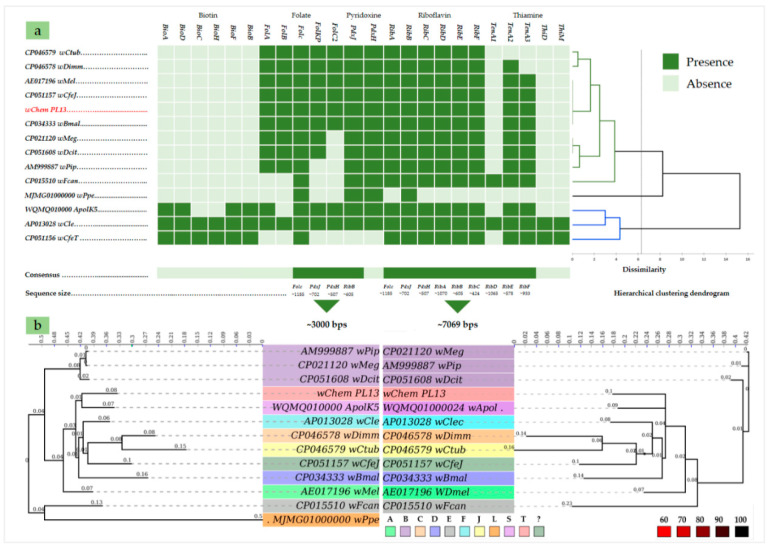
(**a**). Comparative analysis of B-vitamins biosynthesis pathways of the *wChem* PL13 strain and the other *Wolbachia* strains. a. Matrix based on the presence/absence of B-vitamins biosynthesis genes from the *wChem* PL13 and the other *Wolbachia* strains. The matrix was organized according to the hierarchical clustering (AHC) of B-vitamins profile among *Wolbachia* strains (left cladogram). (**b**). Maximum likelihood phylogenies based on 3000 bps (right phylogram) and 7069 bps (left phylogram) using respectively K81uf (+G) [44] and GTR (+G) [45] substitution models. Likelihoods values were −16949.78 and −36573.10 respectively. Values above branches indicate the length of each branch’s, while the axis showed the global distance observed throughout the trees. Color codes indicating the *Wolbachia* supergroup (label) and the bootstraps percent’s (branches).

The inspection of *wChem* PL13 genome revealed B-vitamins synthetic pathways commonly present in the most *Wolbachia* genomes. This include the complete pathway for riboflavin (vitamin B2) and for folate (vitamin B9) with a partial pathway for both pyridoxine (vitamin B6) and thiamine (vitamin B1). Unlike *Wolbachia* of *C. lectularius*, the biotin (vitamin B7) biosynthesis pathway was completely absent in the genome of the *wChem* PL13 strain (Figure 4a).

Both datasets based on gene involved in B-vitamins biosynthesis produced a close topology and very similar posterior bootstrap values. Insect-associated *Wolbachia* from the A, B and E supergroups as well as the supergroup L from the non-filarial nematode had an earliest divergence compared to the filarial and the other insect-associated *Wolbachia*. However, the *wChem* PL13 strain and the supergroup T appeared to be a monophyletic sister with the clade regrouping the supergroup F from *C. lectelarius* and C, D and J supergroups from filarial nematodes (Figure 4b), suggesting a less older B-vitamins biosynthesis genes compared to those from the other insect-associated *Wolbachia* (e.g., A, B and E supergroups).

### 2.4. Comparative Phylogenies and Placement of Wolbachia sp. Strain wChem PL13 in the Wolbachia T Supergroup

Together, the SLST based on the 16S and the WSP genes, the MLST based on the ten selected genes (16S, 23S, *GroL*, *rpoB*, *gatB*, *coxA*, *dnaA*, *fbpA*, *puuA* and *nusA*) as well as the genome-based phylogeny allowed the comparison of the *wChem* PL13 strain with all known *Wolbachia* supergroups except for G, Q and P supergroups, where the suitable dataset were not available. However, the blast comparison of the 16S gene from the *wChem* PL13 strain with *Wolbachia* of *Diaea* sp. (AY486069) supergroup G [46], *Wolbachia* of *Torotrogla cardueli* (KP114100) supergroup Q and *Wolbachia* of *Torotrogla merulae* (KP114099) supergroup P [47], revealed an identity-query cover of 97.77–53%, 97.72–41% and 98.54–41% respectively, which is lower than the 98.7% threshold used to discriminate bacterial species [48].

**Figure 5:**
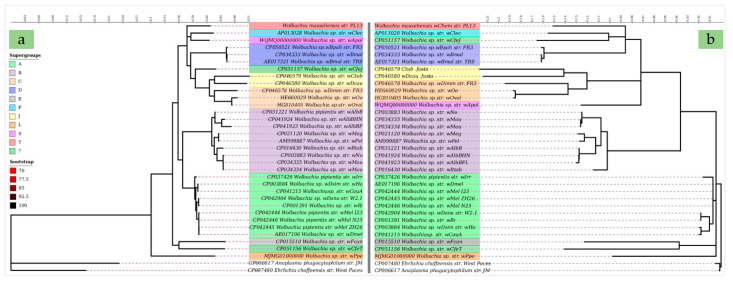
Comparative phylogenies showing the position of the *wChem* PL13 strain among the other *Wolbachia* supergroups. (**a**). IQTREE based on ML method with 1000 bootstraps from the concatenated ten selected genes using GTR (+F+R3) substitution model [49]. Outgroup taxons “*Ehrlichia chaffeensis* (CP007480) and *Anaplasma phagocytophilum* (CP006617)” are drawn at root. Log-likelihood of consensus tree is −88934.819989. (**b**). Genome based phylogeny generated using the FastTree Version 2.1.10, double precision (No SSE3) [50]. The tree was rooted using Jukes-Cantor Joins model with 1000 local boots. The nearest-neighbor interchange (NNI) and the subtree pruning and regrafting (SPR) (2 rounds range 10) were used for the tree rearrangement. The Top Hits was 1.00 * sqrtN.

All cladograms constructed from the SLSTs (Figure S4a,b), MLST (Figure 5a) and genome-based phylogeny (Figure 5b) supported the divergence of the *wChem* PL13 strain from known *Wolbachia* supergroups. Notably, both MLST and genome-based phylogenies produced similar topologies within the same clade and very similar bootstrap values. The only topology differences between trees based on MLST and the *Wolbachia* whole genome datasets were the varying positions of *wCfeJ*, an undescribed *Wolbachia* supergroup from *C. felis* (CP051157) and *wApolK5*, *Wolbachia* supergroup S from *A. politus* (WQMQ00000000), but otherwise no conflicts in *Wolbachia* clade topologies were observed (Figure 5a,b).

### 2.5. Description of Wolbachia massiliensis sp. nov.

From all descriptive results taken together, the *wChem*-PL13 isolated from the bedbug *C. hemipterus* strain constitutes a divergent *Wolbachia* strain. Genotypic profile based on the 16S and the WSP phylogenies, the MLST combining the 16S and 23S rRNA, rpoB, GroL, CoxA, DnaA, fbpA, Asn/Gln, gatB, NusA and PuuA and the genome-based phylogeny as well as the taxo-genomic features delineated a distinct species, clearly different from all other recognized *Wolbachia* strains. We propose the name *Wolbachia masseliensis* sp. nov. designated *wChem*-PL13 strain.

*Wolbachia masseliensis* (mas.si’ li.en.sis. L. gen. adj. massiliensis, from Massilia, the Latin name of Marseille, France, where the organism was first grown, identified and characterized). Since the current *Wolbachia* supergroup classification system is yet be revisited [29], we maintain the notion of supergroup at a strain level and we propose a new supergroup T with a type strain *Wolbachia masseliensis* sp. nov. strain *wChem*-PL13. The known host of this bacterium is *Cimex hemipterus*, a wild strain from Senegal. This isolate has been deposited in the strain collection CSUR (Collection de Souches de l’Unité des Rickettsies WDCM 875) under the accession number CSURP2929. The complete genome sequence of *W. masseliensis* is available in GenBank: Bio Project PRJNA663644; Bio Sample: SAMN16175503 and genome accession number CP061738.

The cells are best visualized by the Diff-Quick staining and appear to be gram negative, small isolated cocci with an average dimension of 570 nm (range: 530 to 615 nm). The bacteria are obligate intracellular and occur inside vacuoles of eukaryotic cells (*A. albopictus* and *D. melanogaster*). The bacteria grow in S2-cell line at 28 °C in Schneider medium supplemented with 10% of decomplemented Bovine Serum Albumin (BSA) and 1% of the combination Penicillin/Streptomycin antibiotics.

## 3. Discussion

In the present study we demonstrate the possibility to use S2 cells for the isolation and maintenance of *Wolbachia*. Our data demonstrated the susceptibility of two arthropod cells derived from *D. melanogaster* and *A. albopictus* mosquito, arthropods naturally infected with well-known *Wolbachia* supergroup A and B, respectively. However, the best bacterial growth was obtained after two weeks on *Drosophila* S2 cell lines. Several cell lines were previously used in the isolation and/or cultivation of *Wolbachia* bacterium including the Aa23 mosquito cell line [51,52], C6/36 cells, another *A. albopictus* cell line and the human embryonic lung (HEL) fibroblast monolayers [52] for wALB13, *Wolbachia* supergroup B from *A. albopictus*. Different cell lines derived from *Ixodes scapularis* and *I. Ricinus* as well as well as the *A. albopictus* cells (AeAl-2) were used for the propagation of three *Wolbachia* strains *wStri*, supergroup A, *wAlbB* supergroup B and *wCfeF* supergroup F from *Laodelphax striatellus*, *A. albopictus* and *C. felis*, respectively [3]. 

Despite the successful propagation of different *Wolbachia* supergroups on different mammalian and insects cell lines, there are no standardized cell line for the co-culture of *Wolbachia*. Our data showed that the infection of up to 97% S2 cells with *Wolbachia* from *C. hemipterus* occurred at 11 days which consist with the results previously obtained on Aa23 mosquito cells, naturally found infected with *Wolbachia* [51]. Since the ability of the S2 cell line to provide a similar *Wolbachia* growth to that obtained on naturally infected cell line, we propose S2 cell line as standard line for *Wolbachia* culture.

It is clear from our results that culture temperature affects the growth of *Wolbachia*, where the best growth was always at 28 °C, the adequate temperature for S2 and C6/36 cell lines. However, several studies shown that the in vivo growth of *Wolbachia* was always in line with the culture temperature of the cell line which varied from 28 °C to 37 °C [3,51,52].

Recently, it was demonstrated that changes to host temperature preference do not alter bacterial load of several A and B-supergroup *Wolbachia* strains. However, hosts infected with A-group *Wolbachia* strains prefer cooler temperatures while those infected with B-group Wolbachia strains prefer a warmer temperature, suggesting that *Wolbachia* strains are differently involved in the host-thermoregulation [53].

Recently, an efficient genome sequencing approach based on probe hybridization enrichment was developed to provide *Wolbachia* genomes directly from their hosts [54]. However, the application of the approach was strongly limited by the amount of bacteria from their hosts, as it was the cases of *wApolK5*, *Wolbachia* supergroup S from *A. politus* (WQMQ00000000) and *wLbra*, *Wolbachia* supergroup D from *Litomosoides brasiliensis* (WQMO00000000) [7,55]. In addition to the manipulation of the bacterium, the standardized protocol for the isolation, culture as well as the purification of *Wolbachia* herein we described, leading to easily obtain enough bacterial DNA which facilitate the genome sequencing.

We obtained the complete genome of *wChem* PL13 strain, *W. massiliensis*, a type strain of new supergroup T from *C. hemipterus* (1,250,060 bp long) which closely mimic the size of *wCle*, *Wolbachia* supergroup F from *C. lectelarius* (1,291,339) and *wDmel*, *Wolbachia* supergroup A from *D. melanogaster* (1,267,782) genomes. However, it seems to be the smaller complete genome from insect-associated *Wolbachia* since the size of complete insect-associated *Wolbachia* genomes ranged between 1, 133,809 bp long and 1,801,626 bp long [6,56]. While it was clearly bigger than those associated to nematodes where the size ranged from 920,122 bp long to 1,080,064 bp long [55,57]. Furthermore, we noted the presence of two sequences coding for phage-like proteins, such as portal, coat transposons, and integrase proteins (Figure S3). *Wolbachia* phage-like proteins were mostly identified in insect-associated *Wolbachia* and in *wPpe*, *Wolbachia* supergroup L from plant parasitic nematode (MJMG01000000), while they were completely absent in filarial-associated *Wolbachia* (Table 1) suggesting *Wolbachia* bacteriophage (WO) infections from the environment of their hosts. Furthermore, the molecular analysis of the prophage coding sequences from *W. massiliensis wChem* PL13, revealed a partial similarity with those encountered in *wDmel*, *Wolbachia* of *D. melanogaster* from supergroup A (AE017196) and *wFcan*, *Wolbachia* of *F. candida* from supergroup E (CP015510). *Wolbachia*-bacteriophage WO relationship was molecularly studied in wasps community [58]. Authors were noted the absence of congruence between WO and host *Wolbachia* as well as WO and insect host, suggesting that the phage WO exchanged frequently and independently within the closed syconium [58]. 

The genome of *W. massiliensis*, *wChem* PL13 strain from the bedbug *C. hemipterus* revealed similar metabolic capacities among the parasitic insect-associated *Wolbachia* from A and B-supergroups. By contrast, the mutualistic bedbug-*Wolbachia* supergroup F from *C. lectularius* [59] closed to the other mutualistic *Wolbachia* supergroups [57] (Figure 4). This emphasis the diversity of *Wolbachia*-host relationship among the bedbugs. Except for the translation COG category [J] which appears to be reduced in the genome of *W. massiliensis* suggesting a strong dependence to its host. Though the lack of information about the distribution of *Wolbachia* as well as their relationship within *C. hemipterus* hosts, which may represent a limitation of our study, the clear-cut distinction between mutualism and parasitism cannot yet be ruled out. Although it is difficult to conclude about the provision of nutritional elements to the host by *W. massiliensis* as long as the biotin pathway was completely absent. The biotin appears to be rare among *Wolbachia* supergroups and was detected only in few insect-associated *Wolbachia* [7]. The complete *Wolbachia* biosynthesis pathway for the biotin was firstly detected in the bedbug *Wolbachia* of *C. lectularius wClec*. In addition to the biotin, *wClec* participates in the host fitness by producing B-vitamins [59,60]. The biotin was latter well studied in the community of *Cimex* and *Paracimex* arthropods using gene-specific PCRs [60]. Authors reported the functional biotin pathways in at least 10 out of 15 studied genera but not in *C. hemipterus* [60]. The absence of *Wolbachia*-biotin production in the bedbug *C. hemipterius* could be compensated by the other symbiotic bacterium. The genetic study of the origin of biotin operons demonstrated that they were acquired via lateral gene transfer presumably from a coinfecting endosymbiont *Cardinium* or *Rickettsia* [59]. The congruence between the phylogenies of B-vitamins operons (i.e., riboflavin, folate and pyridoxine) consistently exhibited similar evolutionary patterns with *Wolbachia* phylogeny. Consequently, it is conceivable, although speculative, B-vitamins synthesis genes are originated from the other symbiotic bacterium within their hosts as it was the case of biotin and thiamine from the bedbug-*Wolbachia* of *C. lectelarius* and the obligate symbiont *Wigglesworthia glossinidia* of tsetse flies [59,61]. 

## 4. Materials and Methods

### 4.1. Source of the Bacterium, Inoculum Preparation and Isolation

The bacterial strain was isolated from wild *Cimex hemipterus* (Fabricius, 1803) collected in Dakar, Senegal (2017). Ten adult bed bugs were morphologically identified on the basis of the following criteria: width/length ratio of the pronotum less than 2, lateral lobes of the pronotum are narrow and hind margins of hemelytral pads are broadly rounded on the inner halves. Once identified, the adult specimens were individually used to isolate the intracellular bacterium using cell co-culture method using the Schneider 2 cell-line (S2) primarily derived from a culture of the late stage (20–24 h old) *Drosophila melanogaster* embryos [62]. This cell line has previously proven to be receptive for several Rickettsiales bacterium such as *Rickettsia assemboensis* and *Rickettsia felis* [63,64]. Briefly, adult bugs *Cimex hemipterus* were rigorously decontaminated by immersing adult bedbug during 5 min into 1% of Sodium Hypochlorite solution (Sigma Aldrich, Saint-Quentin-Fallavier, France followed by rinsing in sterile water and immersing into 70% ethanol and sterile water rinsing again. Each specimen was manually crashed in 1 mL of Schneider medium (Sigma Aldrich, Saint-Quentin-Fallavier, France) to generate the bacterial inoculum. *Wolbachia* cell co-culture was inoculated in the shell vial tubes containing 1 mL of S2 culture as described elsewhere [65,66]. Culture media consisted of a Schneider medium supplemented with 10% of decomplemented Bovine Serum Albumin (BSA) (Sigma Aldrich, Saint-Quentin-Fallavier, France) and 1% of the combination Penicillin/Streptomycin (Sigma Aldrich, Saint-Quentin-Fallavier, France) antibiotics to avoid ubiquitous bacterial contamination. The mixture was sterilized using 0.2 µm filtration and was then kept at 4 °C until use. The infection was performed using 200 µL of the bacterial inoculum derived from adult *C. hemipterus*. One hour of centrifugation at 4000 rpm at 28 °C was performed to increase *Wolbachia*-cell adhesion. Shell vials of infected cultures were kept at 28 °C. Isolation success was assessed using Diff-Quick™ staining (Dade Behring, Marburg, Germany) each 7 days followed by sequencing of the 16S RNA gene [67]. During the isolation period, the maintenance of cell culture was performed by a partial renewal of culture medium each 7 days. Shell vials were centrifuged at 3000 rpm at 28 °C, then a half of the supernatant (500 µL) was replaced by a fresh medium under sterile conditions.

### 4.2. Morphological Characterization and Scanning Electron Microscopy

Infected cells were cytocentrifuged for staining with Gimenez and Diff-Quick (Dade Behring, Marburg, Germany) and were then examined under light microscope Leica^®^ DM LB2.

For the electron microscopy, 200 µL of 11 old days PL13-S2 co-culture were centrifuged for 15 min at 3000 rpm, then the supernatant was removed, and the pellet was fixed using 2.5% glutaraldehyde (Sigma Aldrich, Saint-Quentin-Fallavier, France) in 0.1 M sodium cacodylate buffer (Sigma Aldrich, Saint-Quentin-Fallavier, France) for 1 h. After fixation, the pellet was rinsed three times with 0.1 M sodium cacodylate (5 min each) to remove residual fixative. The graded ethanol concentrations (25% for 5 min; 50% for 5 min; 70% for 5 min; 85% for 5 min; 95% for 5 min (twice); 100% ethanol for 10 min (three times) was used for sample dehydration. Finally, the pellet was incubated for 5 min in an ethanol/Hexamethyldisilazane (Sigma Aldrich, Saint-Quentin-Fallavier, France) (1:2) mixture, then in pure HMDS. The mixture was cytocentrifuged for 5 min at 2000 rpm and the glass slide allowed to air dry for 30 min before observation. The examination was performed using a TM4000 Plus^TM^ (Hitachi, Tokyo, Japan) scanning electron microscope operated at 10 kV in BSE mode at magnifications ranging from X200 to X3000.

### 4.3. Cell Co-Culture Standardization and Wolbachia Production

Once the isolation success was confirmed by optical microscopy and 16S rRNA sequencing, the infected cells were transferred into 15 mL cap flasks for the maintenance of isolated strain. Medium changes were performed each 15 days by centrifugation for 15 min at 3000 rpm at 28 °C, then the supernatant of the old medium was removed and replaced with the same volume of fresh medium. The mixture was subjected to serial gentle repeated pipetting until homogenization, then transferred to sterile cap flasks and maintained at 28 °C.

To optimize *Wolbachia* cell co-culture, another arthropod cell line (C6/36) derived from *Aedes albopictus* mosquitoes was investigated. C6/36 cell line (CRL-1660; American Type Culture Collection) was maintained in 75 mL cap flasks containing the Leibowitz-15 medium with L-glutamine and L-amino acids (Gibco™, Thermo Fisher Scientific, Inc., Waltham, MA, USA), 5% (vol/vol) fetal bovine serum, and 2% (vol/vol) tryptose phosphate (Gibco™, Thermo Fisher Scientific, Inc., Waltham, MA, USA) at 28 °C. One mL of a C6/36 rich cell culture was transferred to shell vial tubes 24 h prior to the infection. *Wolbachia* inoculum was obtained from a lysate of the *Wolbachia* S2 cells following a serial aspiration-injection into 50 mL falcon using a fine needle syringe. The inoculum of 200 µL was used for the infection of C6/36 cells previously prepared in shell vial tubes as described above. The receptivity of C6/36 was first checked at day 15 post-infection using Diff-Quick™ staining. Once the infection was confirmed, the infected cells were transferred into 75 mL cap flasks containing 14 mL of 1 old day of C6/36 cell culture.

Bacterial growth was investigated from both infected cells under two different temperatures: 28 °C and room temperature. Three cap flasks per each cell-line for each condition were followed for one month on the weekly schedule using the pan-*Wolbachia* 16S rRNA qPCR [All-Wol-16S qPCR] [68]. The repeated measures Analysis of Variance (ANOVA) was used to evaluate the effect of both temperature and cell lines on bacterial growth, while a pairwise comparison using Tukey test was performed to evaluate whether condition is more suitable. Statistical analysis were performed using XLSTAT Addinsoft version 4.1 (XLSTAT 2019: Data Analysis and Statistical Solution for Microsoft Excel, Paris, France).

### 4.4. Purification of the Bacterium

S2 cells infected with the bacterium were produced in a total volume of 75 mL spread over three 150 cm^2^ cell culture flasks. The infection rate of 97% was obtained at day 11 post-inoculation with the bacterium. Infected cells were harvested from the three flasks, then were checked for the presence of bacterial and fungal contaminations using both the Diff-Quick™ staining and the bacterial 16S rRNA sequencing. The suspension was subjected to three cycles of sonication of 1 min at 20 Hz, after which unlysed cells were removed by centrifugation at 500 rpm for 10 min. The supernatant containing the bacterium was layered onto a density gradient solution of 15% weight/volume (wt/vol) sucrose in phosphate-buffered saline (PBS, Sigma Aldrich, Saint-Quentin-Fallavier, France). After centrifugation at 9000× *g* for 45 min at 4 °C, the bacterium-containing pellet was resuspended in 2 mL of PBS and carefully layered onto a 20 to 45% (wt/vol in PBS) step density gradient. This gradient was subjected to centrifugation at 9000 rpm for 45 min at 4 °C; and the bacteria were harvested and washed twice in PBS, resuspended in sterile distilled water in the smallest possible volume, and then frozen at −80 °C. At each time point, the pan-*Wolbachia* 16S rRNA qPCR and the Diff-Quick™ staining were performed to assess bacterial load.

### 4.5. Genome Sequencing and De Novo Assembly

Genomic DNA was extracted from 200 µL of purified bacterium. The extraction was performed using QIAGEN DNA tissues kit (QIAGEN, Hilden, Germany) following the manufacturer’s recommendations. An additional lysis step was applied prior to the extraction procedure using a pre-treatment by lysozyme incubation with buffer G2 and proteinase K for 2 h at 37 °C. The extracted gDNA was eluted in a total volume of 50 µL. Genomic DNA (gDNA) was quantified by a Qubit assay with the high sensitivity kit (Life technologies, Carlsbad, CA, USA); the concentration was equal to 42.86 ng/µL. The DNA was diluted at 1ng as input to prepare the paired end library. The gDNA was barcoded in order to be mixed with other genomic projects with the Nextera Mate Pair sample prep kit (Illumina). The purification on AMPure XP beads (Beckman Coulter Inc. Waltham, MA, USA) was performed prior to the normalization of the libraries on specific beads according to the Nextera Mate Pair Illumina guide. Automated cluster generation and sequencing run with dual index reads were performed in a single 39 h run in a 2 × 251-bp format. Within this run, a total of 190,631 reads were generated and were quality-checked using FastQC, trimmed using Trimmomatic version 0.36.624 and assembled in seventy-eight (78) scaffolds using the SPAdes version 3.5.0 sofware25. The option “careful” was used to reduce the number of mismatches and short indels. Default Nanopore technology (Oxford Nanopore Technologies Ltd., Oxford, United Kingdom) was used by 1D genomic DNA sequencing on the MinION device using the SQK-LSK108 kit. The library was constructed from 1.5 µg of genomic DNA without fragmentation and end repair. Adapters were ligated to both ends of genomic DNA. After purification on AMPure XP beads (Beckman Coulter Inc. Waltham, MA, USA), the library was quantified by a Qubit assay with the high sensitivity kit (Life Technologies, Cat. no. Q32856) and loaded on the flow cell via the SpotON port. A total of 466 active pores were detected for the sequencing and the workflow WIMP was chosen for sequence analysis. Adapter trimming, quality filtering and error correction of all sequencing raw data analyzed here were performed using the Trimmomatic program (version 0.36). Finally, mean read quality was 11.2 (median = 11.9). A total of 78,865 reads were generated with a mean length of 1,392.9 (median 762) and an N50 read length of 2493, which corresponds to 109,847,620 pair bases (pb).

### 4.6. Comparative Genomic Analyses and Annotation

First, the PL13 strain and eighteen other *Wolbachia* genomes were annotated using DFAST [69,70]. Numbers of orthologous proteins shared between genomes were visualized using Circos [71]. Nine *Wolbachia* genomes including *Wolbachia* supergroup A, B, C, D, E, F, L, S and an undescribed supergroup from flea [72] were selected for genomic comparisons. The circular map of the complete chromosome of PL13 strain was generated using GCviewer (http://stothard.afns.ualberta.ca/cgview_server/). Annotation, completeness, and contamination values were estimated for the PL13 strain as well as the nine selected *Wolbachia* genomes using DFAST [69,70]. The presence of prophage regions was predicted using PHASTER [73]. Orthologous Average Nucleotide Identity (Ortho-ANI) [74] was used to evaluate the degree of genomic similarity between the PL13 strain and the other *Wolbachia* genomes. The pan genome distribution was evaluated using Raory software [75]. 

Additionally, the pervious dataset was enriched by adding four genomes representing the supergroup B, J and another undescribed supergroup from flea [72]. The Genome-to-Genome Distance Calculator Web service was used within the formula 2 to calculate the Digital DNA-DNA hybridization (dDDH) [76,77]. The probability that an intergenomic distance yielded a dDDH larger than 70%, representing a novel species-delimitation threshold [78]. Similarly, the prodigual was used for prediction in the Open Reading Frame (ORF) with the default settings [79]. Deviations in the sequencing regions predicted by ORFs have been excluded. BlastP was used to predict the bacterial proteome (*E* value of 1e03, coverage of 0.7 and percent identity of 30) according to the Orthological Group (COG) database [80]. In the absence of match within the COG database, the BlastP was performed against the GenBank nr database [81] within an *E* value of 1e03, coverage of 0.7 and 30% of identity. On the other hand, when the length of the sequence is less than 80 amino acids (aa), an *E* value of 1e05 has been used. The hmmscan analysis tool [82] was used on the PFAM-A and PFAM-B domains. The assigned COGs for each genome were ordered in 26 different categories and were then compared using the Agglomerative Hierarchical Clustering (AHC) analysis. KEGG Orthology (KO) assignments was performed for the 14 *Wolbachia* genomes using KASS (KEGG Automatic Annotation Server) [83]. The KASS analysis was performed using BBH (bi-directional best hit) method. The assigned KO number were ordered in 177 different pathways and were then assessed using the heat map (OMICS) method. The analysis excluded all pathways that having a variance lower than 0.25. All statistical analysis were performed using XLSTAT Addinsoft version 4.1 (XLSTAT 2019: Data Analysis and Statistical Solution for Microsoft Excel, Paris, France).

### 4.7. B-Vitamins Biosynthesis Pathway

Genes involved in the biosynthesis of B-vitamins such as the biotin (vitamin B7), riboflavin (vitamin B2), pyridoxine (vitamin B6), folate (vitamin B9) and thiamine (vitamin B1) synthesis genes were retrieved from the bedbug-*Wolbachia* (*wClec*) [59] and were used to search their homologous from the other *Wolbachia* genomes using Blastn. B-vitamin profile of selected *Wolbachia* was assessed using the AHC analysis according to the presence/absence of genes. To test whether *Wolbachia* B-vitamins synthesis genes are conserved among *Wolbachia* supergroups and have the same ancestors, we phylogenetically compared all B-vitamins synthesis genes presumably common within *Wolbachia* supergroups. This include two datasets based on the following genes: (i) *FolC*, *PdxJ*, *PdxH* and *RibB* from 13 *Wolbachia* genomes including *wPpe*, *Wolbachia* supergroup L from the earliest *Wolbachia* host *P. penetrans* (MJMG01000000) [11] and (ii) *FolC*, *PdxJ*, *PdxH*, *RibA*, *RibB*, *RibC*, *RibD*, *RibE* and *RibF* from the same genome dataset except for wPpe which was excluded because it lacking for the complete gene datasets. Genes from each dataset were aligned using MAFFT [84] and concatenated within Seaview [85]. Best fit phylogenetic model was selected for each dataset and the maximum likelihood phylogeny was performed using 1000 bootstraps replicates. Molecular phylogenetic analyses were conducted on Topali v.2 software [86].

### 4.8. Comparative Phylogenies and Taxonomy

First, two single locus sequence typing (SLST) phylogenies were performed on the basis of the 16S rRNA and *Wolbachia* surface protein (WSP) genes. Briefly, a full-length sequence from both genes were retrieved from the annotated genome of the PL13 strain. The 16S sequences were aligned against the representative members of fifteen (A, B, C, D, E, F, H, I, J, K, L, M, N, O, and S) and two undescribed *Wolbachia* supergroups. While the WSP sequence of the PL13 strain was aligned against the representative members of ten *Wolbachia* supergroups (A, B, C, D, E, F, J, R and S) and two undescribed supergroups. All alignments were performed using the ClustalW application within Bioedit v.7.2.5. [87]. The Akaike Information Criterion (AIC) option in MEGA6 [88] was used to establish the best nucleotide substitution model for the 16S sequence alignment. The Kimura 2-parameter model (+G) [89] was selected and the maximum likelihood (ML) phylogenetic inference was used with 1000 bootstrap replicates to generate the 16S tree in MEGA6 [88]. The 16 sequences from *Ehrlichia chaffeensis* (CP007480) and *Anaplasma phagocytophilum* (CP006617) were used as out groups to root the tree. The WSP phylogeny was inferred using ML method with 1000 bootstrap replicates on IQ-TREE [49]. The most appropriate model of evolution was evaluated by Modelfinder (implemented as functionality of IQ-TREE). The analysis was performed on Galaxy Australia server (https://usegalaxy.org.au/).

Genome-based phylogeny was performed for *Wolbachia* PL13 and 32 other complete/draft genomes of *Wolbachia* including nine *Wolbachia* supergroups: A, B, D, E, F, J, L and S and two undescribed *Wolbachia* to belong to any supergroup [72]. *Anaplasma phagocytophilum* (CP006617) and *Ehrlichia chaffeensis* (CP007480) genomes were included as outgroups. All genomes were aligned using the global alignment with conserved columns and gaps on Scapper software (https://github.com/tseemann/scapper). The FASTTREE [50] was builded using ML method. GTR + CAT model was selected and the pseudocounts option was activated. This latter is recommended for highly gapped/fragmentary sequences [50]. The tree was rooted by turning on maximum-likelihood option at 1000 replicates.

Additionally, a multi loci sequence typing (MLST) phylogeny was performed on the basis of ten selected genes including: both the 16S and 23S ribosomal RNAs, DNA-directed RNA polymerase subunit beta (*rpoB*), 60 kDa chaperonin (*GroL*), cytochrome c oxidase subunit 1 (*CoxA*), chromosomal replication initiator protein (*DnaA*), fructose-bisphosphate aldolase (*fbpA*), aspartyl/glutamyl-tRNA(*Asn/Gln*) amidotransferase subunit B (*gatB*), transcription termination/antitermination protein (*NusA*) and gamma-glutamylputrescine synthetase (*PuuA*). The choice of these loci was conform to the standard loci requirements for an MLST system [90]. These genes were retrieved from the 35 genomes after annotation on DFAST. MAFFT alignment [84] was performed and sequences were then merged using Seaview [85]. ML phylogeny was performed with IQ-TREE [49] using 1000 bootstrap replicates. All phylograms from the SLST, MLST and FASTRTREE phylogenies were edited by iTOL v4 software [91].

## 5. Conclusions

Our results emphasize the usefulness of the S2 cells as a suitable line for the isolation and the propagation of *Wolbachia*. Thereby, the standardized procedure herein we proposed provided sufficient material for genome sequencing and other manipulation. This may help to resolve problems related to the direct sequencing of wolbachial genomes from their hosts [7,54] as well as to cultivate the previously uncultivated *Wolbachia* (e.g., filaria-associated *Wolbachia*) since the S2 line allowed the successful culture of the PL13 strain in record time (11 days) despite the reduction of translational machinery of this bacteria.

Genomic and metabolic features that emphasize both nutritional-mutualistic and parasitic relationship between *wChem* PL13 and its host. However, the clear-cut distinction between mutualism and parasitism of this strain cannot be yet ruled out and further studies are needed.

These features provide the platform for the feature research to understand *Wolbachia*-host interaction. By combining bacterial isolation and taxo-genomic descriptions may ultimately assist in the quest to classify *Wolbachia* in multiple species as is the case of the other Rickettsiales bacterium such as *Rickettsia* spp. *Anaplasma* spp., and *Ehrlichia* spp.

## Figures and Tables

**Table 1:** General features of *wChem* PL13 and other *Wolbachia* genomes.

Strain Information	*wChem* PL13	*wClec*	*wDmel*	*wPip*	*wFcan*	*wApol*	*wCfeJ*	*wBmal*	*wDimm*	*wPpe*
Host type	Insects						Nematodes			
*Wolbachia* host	*C. hemipterus*	*C. lectularius*	*D. melanogaster*	*C. quinquefasciatus*	*F. candida*	*A. politus*	*C. felis*	*B. malayi*	*D. immitis*	*P. penetrans*
Supergroup	New supergroup “T”	F	A	B	E	S	Undescribed	D	C	L
**Genome features**										
Accession Number	CP061738	AP013028	AE017196	AM999887	CP015510	WQMQ00000000	CP051157	CP034333	CP046578	MJMG01000000
Total length (bp)	1,291,339	1,250,060	1,267,782	1,482,455	1,801,626	1,445,964	1,201,647	1,080,064	920,122	975,127
No. of contigs	1	1	1	1	1	373	1	1	1	12
GC content (%)	35.4	36.3	35.2	34.2	34.4	35.6	35.6	34.2	32.7	32.2
N50	1,291,339	1,250,060	1,267,782	1,482,455	1,801,626	5741	1,201,647	1,080,064	920,122	9,555
Gap ratio (%)	0.326793	0.0	0.0	0.006746	0.0	0.0	0.0	0.0	0.0	0.13547
No. of CDSs	1194	1226	1211	1395	1591	1546	1045	1017	709	939
No. of rRNA	3	3	3	3	3	3	3	3	3	3
No. of tRNA	32	34	34	34	35	39	34	34	34	35
No. of CRISPRS	0	0	0	0	0	0	0	0	0	0
Coding ratio (%)	78.5	77.3	81.6	84.4	86.9	66.1	82.3	70.1	70.7	84.8
Completeness (%)	98.00	98.00	98.73	99.45	97.27	95.89	98.36	99.09	98.00	93.32
Contamination (%)	0.36	0.36	0.00	0.00	1.55	19.67	0.36	0.00	0.00	2.73
No. of prophage	2	3	3	4	6	2	0	0	0	1

**Table 2:** dDDH values of the *wChem* PL13 comparatively to the other *Wolbachia* supergroups.

Strain	*Wolbachia* Host	Accession Number	DDH	Distance	Prob. DDH ≥ 70%	G + C Difference	Model C.I.
*wPpe*	*P. penetrans*	MJMG01000000	19.8	0.2216	0	3.21	[17.6–22.2%]
*Ctub*	*C. tuberocauda*	CP046579	23	0.1901	0	3.09	[20.7–25.5%]
*WCfelT*	*C. felis*	CP051156	23.6	0.1853	0	0.19	[21.3–26%]
*wFcan*	*F. candida*	CP015510	24.1	0.1809	0.01	1.02	[21.8–26.6%]
*wDimm*	*D. immitis*	CP046578	24.8	0.1757	0.01	2.67	[22.5–27.3%]
*wCmeg*	*C. megacephala*	CP021120	27	0.1599	0.03	1.42	[24.7–29.5%]
*wDcit*	*D. citri*	CP051608	27	0.1604	0.03	1.38	[24.6–29.4%]
*wPip*	*C. quinquefasciatus*	AM999887	27.1	0.1598	0.03	1.18	[24.7–29.5%]
*wCfeJ*	*C. felis*	CP051157	27.8	0.155	0.04	0.2	[25.4–30.3%]
*wBmal*	*B. malayi*	CP034333	28.4	0.1512	0.05	1.19	[26–30.9%]
*wDmel*	*D. melanogaster*	AE017196	29.3	0.1462	0.08	0.14	[26.9–31.8%]
*wClec*	*C. lectularius*	AP013028	29.3	0.1462	0.08	0.88	[26.9–31.8%]
*wApolK5*	*A. politus*	WQMQ00000000	30	0.1423	0.1	0.23	[27.6–32.5%]

## Supplementary Materials

The following are available online at https://www.mdpi.com/1422-0067/21/21/8064/s1.
