# Supplementary material for: An Earliest Endosymbiont, Wolbachia massiliensis sp. nov., Strain PL13 from the Bed Bug (Cimex hemipterus), Type Strain of a New Supergroup T
Source: Int J Mol Sci. 2020 Oct 29;21(21):8064. doi: 10.3390/ijms21218064 (PMC7662661; doi:10.3390/ijms21218064)
Supplement: Supplementary file 1 [file ijms-21-08064-s001.zip › Table S1.docx]

**Table S1:** Weekly follow-up of bacterial growth using qPCR Ct values.

| **Culture conditions** | | **Week 0** | **Week 1** | **Week 2** | **Week 3** | **Week 4** |
| --- | --- | --- | --- | --- | --- | --- |
| **28°C** | **S2** | 33.6 | 28.9 | 27.6 | 23.5 | 16.9 |
|  | **C6/36** | 32.6 | 29.5 | 29.3 | 25.3 | 19.9 |
| **Room Tm** | **S2** | 33.6 | 31.0 | 29.6 | 25.7 | 20.6 |
|  | **C6/36** | 32.6 | 31.5 | 29.8 | 27.1 | 22.8 |
| **Statistics** | ANOVA LS model | R²=0.124/ Pr > F=0.552/ | R²=0.44/ Pr > F=0.074 | R²=0.47/ Pr > F=0.058 | R²=0.886/ Pr > F<0.0001 | R²=0.952/ Pr > F<0.0001 |
|  | 28°C vs room Tm (Pr > Diff) | 1.000 | 0.03 | 0.049 | <0.0001 | <0.0001 |
|  | S2 vs C6/36 (Pr > Diff) | 0.289 | 0.54 | 0.13 | 0.001 | <0.0001 |
