# Supplementary figures and images for: An Earliest Endosymbiont, Wolbachia massiliensis sp. nov., Strain PL13 from the Bed Bug (Cimex hemipterus), Type Strain of a New Supergroup T
Source: Int J Mol Sci. 2020 Oct 29;21(21):8064. doi: 10.3390/ijms21218064 (PMC7662661; doi:10.3390/ijms21218064)

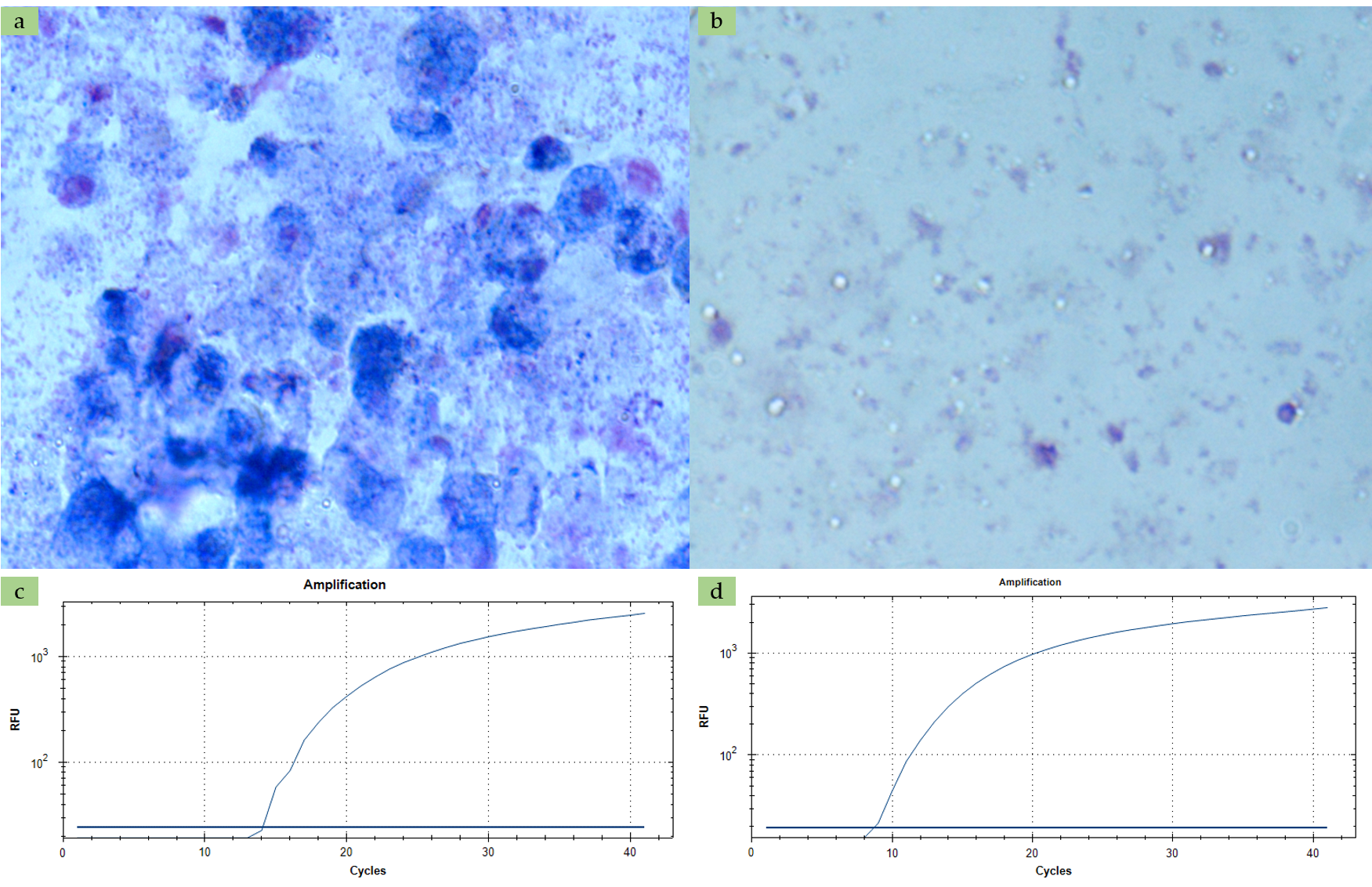

Supplement: Supplementary file 1 [file ijms-21-08064-s001.zip › Figure S1.tif]

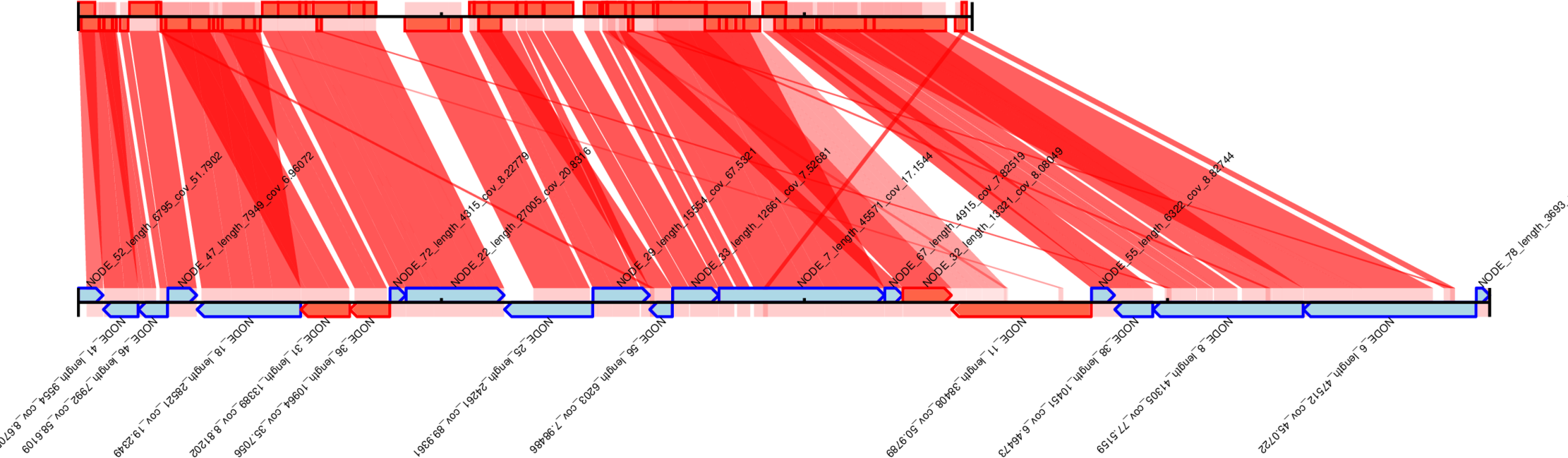

Supplement: Supplementary file 1 [file ijms-21-08064-s001.zip › Figure S2.tif]

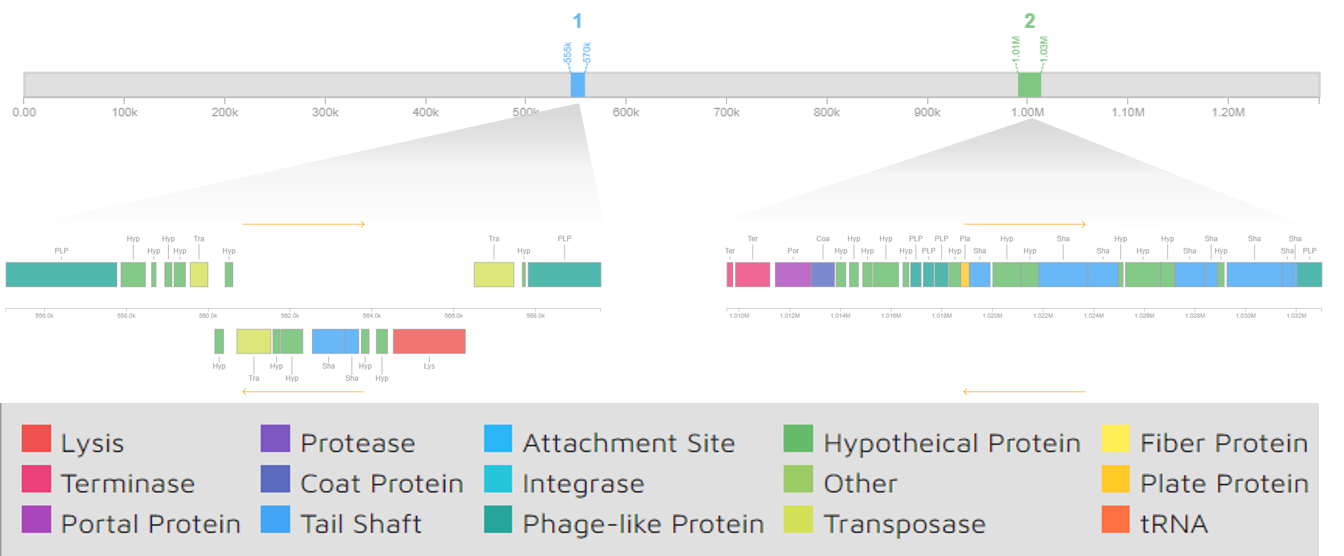

Supplement: Supplementary file 1 [file ijms-21-08064-s001.zip › Figure S3.tif]

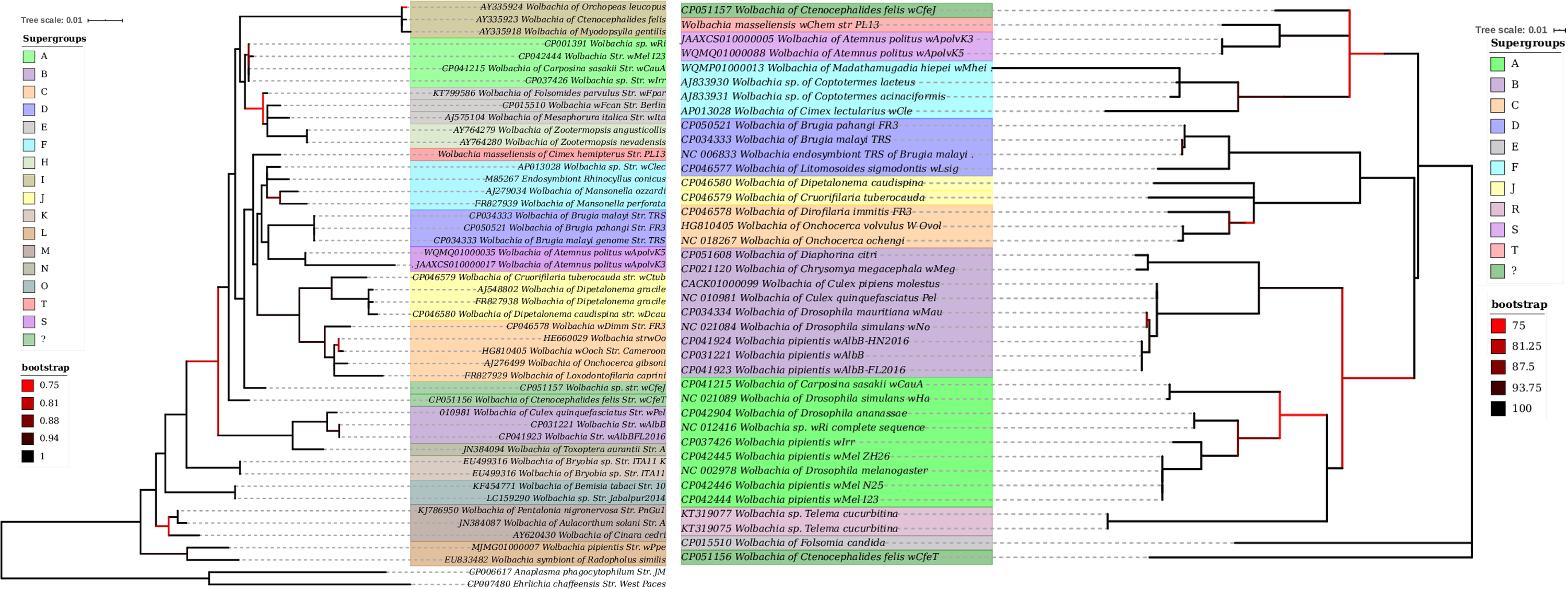

Supplement: Supplementary file 1 [file ijms-21-08064-s001.zip › Figure S4.tif]
